# No association between temperature and deaths from cardiovascular and cerebrovascular diseases during the cold season in Astana, Kazakhstan – the second coldest capital in the world

**DOI:** 10.3402/ijch.v71i0.19769

**Published:** 2012-12-17

**Authors:** Andrej M. Grjibovski, Nassikhat Nurgaliyeva, Aliya Kosbayeva, Bettina Menne

**Affiliations:** 1 Department of International Public Health, Norwegian Institute of Public Health, Oslo, Norway; 2 International School of Public Health, Northern State Medical University, Arkhangelsk, Russia; 3 Medical University of Astana, Astana, Kazakhstan; 4 WHO office in Kazakhstan, Astana, Kazakhstan; 5 WHO European Centre for Environment and Health, Bonn Office, Bonn, Germany

**Keywords:** cold, apparent temperature, mortality, hypertensive disorders, coronary heart disease, cerebrovascular diseases

## Abstract

**Background:**

Several European and North American studies have reported associations between cold temperatures and mortality from diseases of the circulatory system. However, the effects of cold vary between the settings warranting further research in other parts of the world.

**Objectives:**

To study associations between temperature and mortality from selected diseases of circulatory system in Astana, Kazakhstan – the second coldest capital in the world.

**Methods:**

Daily counts of deaths from hypertensive diseases (ICD-10 codes: I10–I15), ischemic heart disease (I20–I25) and cerebrovascular diseases (I60–I69) among adults 18 years and older in Astana, Kazakhstan, during cold periods (October–March) in 2000–2001 and 2006–2010 were collected from the City Registry Office. Associations between mortality and mean apparent temperature and minimum apparent temperature (average for lags 0–15) were studied using Poisson regression controlling for barometric pressure (average for lags 0–3), wind speed and effects of month, year, weekends and holidays. Analyses were repeated using mean and minimum temperatures.

**Results:**

Overall, there were 320, 4468 and 2364 deaths from hypertensive disorders, ischemic heart disease and cerebrovascular diseases, respectively. No significant associations between either mean, mean apparent, minimum or minimum apparent temperatures were found for any of the studied outcomes.

**Conclusions:**

Contrary to the European findings, we did not find inverse associations between apparent temperatures and mortality from cardiovascular or cerebrovascular causes. Factors behind the lack of association may be similar to those in urban settings in Siberia, that is, centrally heated houses and a culture of wearing large volumes of winter clothes outdoors. Further research on the sensitivity of the population in Kazakhstan to climatic factors and its adaptive capacity is warranted.

Deleterious effects of exposure to cold on human health are well recognised (1). Inverse associations between air temperature and both overall and cause-specific mortality, including cardiovascular, cerebrovascular and respiratory causes, have been observed in many European cities (2). However, the effect of cold on cardiovascular mortality varies between settings (2) warranting replication of the findings in other parts of the world. We studied associations between cold and mortality from selected diseases of the circulatory system in the second coldest capital in the world – Astana, Kazakhstan.

## Methods

According to the *Köppen-Geiger classification*, Astana (population 709 K, 2010) is located on the border between a humid continental and a semi-arid climate and has cold winters and warm summers (3) with average temperatures of −17.3°C and 20.2°C for January and July, respectively. Daily counts of deaths from hypertensive diseases (ICD-10 codes I10–I15), ischemic heart disease (ICD-10 codes I20–I25) and cerebrovascular diseases (ICD-10 codes I60–I69) among adults for periods from October through March 2000–2001 and 2006–2010 were obtained from the City Registry Office. Mean and minimum apparent temperatures were calculated as described in detail elsewhere (2) using the data on mean and minimum daily temperatures and humidity from the Kazakhstani Hydrometerological Service (Kazhydromet). Apparent temperatures represent discomfort indices and are more appropriate for studies on the effects of cold since they combine the effect of temperature and humidity. However, we repeated all analyses using mean and minimum daily temperatures. Data on barometric pressure and wind speed were also obtained from Kazhydromet.

First, a curvilinear relationship between temperature and outcomes was modelled by fitting cubic splines with knots spaced every 5°C followed by fitting hockey stick models to assess threshold values for temperature (2). These analyses revealed that the relationships between the temperature and all the outcomes were linear across the whole temperature spectrum. Associations between temperatures and daily counts of deaths were quantified using first-order autoregressive Poisson regression. Given that the effect of cold and barometric pressure can be delayed, average temperature for the current day and the previous 15 days average barometric pressure for the 85 current day and the previous 3 days were used as in (2). Information regarding the month, year and holidays were included in the models as dichotomous variables while wind speed and barometric pressure were used as continuous variables.

## Results

Median values for mean and minimum apparent temperatures for the studied period were 7.7°C and −10.3°C, respectively. No significant associations between daily death counts and either mean or minimum apparent temperatures for any of the studied outcomes were observed (Table I). Additional analyses using mean and minimum temperatures yielded similar results.

**Table I:** Associations between daily counts of death from sel`es (average for the current day and previous 15 days)

				Changes in daily mortality per 1°C		
				
					95% Confidence Intervals	
					
Cause of death	Gender	Number of deaths	Temperature	% Change*	Lower limit	Upper limit
Hypertensive disorders (IDC10 codes: I10–I15)	Men	188	Mean apparent T	−3.4	−10.7	3.9
			Minimum apparent T	−4.4	−13.1	4.2
	Women	132	Mean apparent T	2.0	−4.5	8.5
			Minimum apparent T	−0.2	−8.0	7.6
	Both genders	320	Mean apparent T	−0.2	−5.1	4.6
			Minimum apparent T	−2.0	−7.7	3.7
Ischaemic heart disease (IDC10 codes: I20–I25)	Men	2296	Mean apparent T	−0.4	−2.6	1.8
			Minimum apparent T	−0.7	−3.2	1.8
	Women	2172	Mean apparent T	0.3	−1.7	2.2
			Minimum apparent T	0.2	−2.0	2.6
	Both genders	4468	Mean apparent T	−0.1	−1.6	1.4
			Minimum apparent T	−0.3	−2.0	1.5
Cerebrovascular diseases (IDC10 codes: I60–I69)	Men	977	Mean apparent T	1.4	−2.2	5.0
			Minimum apparent T	1.8	−2.4	6.0
	Women	1388	Mean apparent T	0.9	−2.1	3.8
			Minimum apparent T	0.3	−3.1	3.7
	Both genders	2364	Mean apparent T	1.1	−1.3	3.4
			Minimum apparent T	0.9	−1.7	3.6

* Adjusted for barometric pressure (average for the current day and previous 3 days), wind speed, month, year, holidays and weekends.

## Discussion

Our results suggest no association between the daily counts of deaths from hypertensive disorders, ischemic health disease or cerebrovascular diseases and temperature in Astana, Kazakhstan. These findings partly contradict the results obtained from 15 European cities (2), where cardiovascular, but not cerebrovascular mortality increased in parallel to a decrease in minimum apparent temperature (2). Moreover, the effect of cold on mortality in Europe was less pronounced in cities with lower winter temperatures (2). Given that the median value for minimum apparent temperature in our study is −10.3°C while the corresponding temperature for the coldest city in the abovementioned study was −5.3°C, our results are not surprising and are further supported by evidence from Yakutsk, Russia – the coldest town in the world – where no effect of low temperatures on all-cause or cardiovascular mortality was found (4). This can be explained by the fact that Astana is a rapidly developing capital of the oil-rich Republic of Kazakhstan, where adaptive capacities to harsh climatic conditions were in place traditionally with a well-established warning system, central heating arrangement with the indoor temperature kept between 20 and 24°C in most of the apartments and public transport infrastructure, combined with a culture of wearing fur or feather clothes and hats when going outdoors. We do not recommend applying our results to much less affluent rural areas of Kazakhstan as the quality of housing is poorer and people are more disadvantaged than in the capital city.

## Conclusions

No association between cold temperatures and mortality from hypertensive disorders, ischemic heart disease and cerebrovascular diseases was observed in Astana, Kazakhstan.
